# Developing evidence-based clinical imaging guidelines for the diagnosis of vertically fractured teeth

**DOI:** 10.1007/s11282-024-00766-2

**Published:** 2024-07-08

**Authors:** Ki-Hong Kim, Jo-Eun Kim, Sam-Sun Lee, Chena Lee, Miyoung Choi, Hwan Seok Yong, Seung Eun Jung, Min-Suk Heo, Kyung-Hoe Huh

**Affiliations:** 1 Department of Oral and Maxillofacial Radiology, School of Dentistry and Dental Research Institute, Seoul National University, Seoul, Korea; 2 Department of Oral & Maxillofacial Radiology, Yonsei University College of Dentistry, Seoul, Korea; 3 Division for Healthcare Technology Assessment Research, National Evidence-Based Healthcare Collaborating Agency, Seoul, Korea; 4 Department of Radiology, Korea University Guro Hospital, Seoul, Korea; 5 Department of Radiology, College of Medicine, Eunpyeong St. Mary’s Hospital, The Catholic University of Korea, Seoul, Korea

**Keywords:** Imaging guideline, Vertical root fracture, Periapical radiography, Cone beam computed tomography, Systematic review

## Abstract

**Objectives:**

This study aimed to develop an evidence-based clinical imaging guideline for teeth suspected with vertical root fractures.

**Methods:**

An adaptation methodology based on the Korean Clinical Imaging Guidelines (K-CIG) was used in the guideline development process. After searching for guidelines using major databases such as Ovid-Medline, Elsevier-Embase, National Guideline Clearinghouse, and Guideline International Network, as well as domestic databases such as KoreaMed, KMbase, and KoMGI, two reviewers analyzed the retrieved articles. The retrieved articles were included in this review using well-established inclusion criteria.

**Results:**

Twenty articles were identified through an online search, of which three were selected for guideline development. Based on these three guidelines, this study developed specific recommendations concerning the optimal imaging modality for diagnosing teeth suspected of vertical root fractures.

**Conclusions:**

Periapical radiography is the preferred method for assessing teeth with mastication-related pain and suspected vertical root fractures. However, if intraoral radiographs do not provide sufficient information about root fractures, a small FOV CBCT may be considered. However, the use of CBCT in endodontically treated teeth is significantly constrained by the presence of artificial shading.

## Introduction

The clinical signs of vertical root fractures (VRF) are similar to those of root canal treatment failure or periodontal disease, making diagnosis difficult [1]. However, after conclusive diagnosis, the prognosis of teeth with VRFs is notably poor [2]. Urgent decision-making is required to prevent further bone loss, which can complicate subsequent reconstructive interventions, often necessitating extraction, root amputation, or implantation [1, 3]. Thus, accurate diagnosis is crucial to avoid unnecessary extraction of treatable teeth [4].

Periapical radiography (PA) is widely used to observe trauma sites with minimal radiation exposure. However, fractures may elude detection if the X-ray beam fails to traverse the fracture line [3]. Further, PA radiography does not provide three-dimensional information about the tooth and surrounding structures, with potential overlap obscuring the VRF detection sensitivity [5].

Given the limitations of conventional imaging modalities in visualizing VRFs, alternative imaging systems, such as cone-beam computed tomography (CBCT), are urgently needed [6]. CBCT imaging enables the precise visualization and evaluation of teeth with VRFs [5, 7]. However, VRF often manifests in endodontically treated teeth, with a reported prevalence ranging from 3.7 to 30.8% [8–11]. The presence of radiopaque materials within the root canal, such as gutta-percha or metallic posts, can reduce the diagnostic validity due to beam hardening and streak artifacts mimicking fracture lines [7]. Despite its advantages, CBCT usage is constrained by its high radiation dose, high cost, and limited availability [3].

Given the intricate nature and importance of VRF, selecting an appropriate imaging modality is paramount for diagnosis. Radiological examinations require stringent justification, ensuring that benefits outweigh risks associated with radiation exposure [12, 13]. To regulate the appropriateness of radiological examinations and procedures, clinical guidelines have been developed to support clinical referrals and decisions [14]. In addition to clinical exams, clinicians made diagnosis through imaging such as periapical, panoramic, and even CBCT, and surgical findings. The use of CBCT is increasingly being used to diagnose VRF, although it has not been precisely studied which imaging modalities are used and to what extent. However, there are no clinical imaging guidelines for VRF (Vertical Root Fracture) in South Korea. This study aimed to develop evidence-based Korean clinical imaging guidelines (K-CIG) for teeth with suspected VRFs.

## Materials and methods

Guidelines were developed through collaboration between the Korean Academy of Oral and Maxillofacial Radiology, Korean Society of Radiology, and National Evidence-Based Healthcare Collaborating Agency. The methodology proposed by Choi et al. [15], and applied by Kim et al. [16] was used to develop evidence-based guidelines. For this purpose, a development committee, working group, and consensus group were established.

### Committee composition

The working group handled the development process by selecting key questions. Members recommended by the professional society were chosen for their comprehensive understanding of clinical practice guideline development and proactive engagement in the developmental stages. The working group was formed by assembling the recommended members.

The development committee, comprising experts in oral and maxillofacial radiology, research methodology, and clinical guideline development, assumed a comprehensive planning role, providing support for the study methodology.

Finally, the consensus group comprised six potential end users of the clinical guidelines nominated by five relevant societies who actively participated in an expert panel survey (utilizing the Delphi method) to review key questions and agree on draft recommendations.

### Definition of a key question

The working group formulated key questions for review by the development committee and consensus group. Each question was meticulously crafted to articulate clear and concise sentences encompassing all components of PICO: patient, intervention, comparator, and outcomes.

Following a thorough review, the final version of the key questions was as follows:

### Search for guidelines

Core databases (Ovid-Medline, Elsevier-Embase, Guideline International Network, and National Guideline Clearinghouse) were searched to identify relevant guidelines. Three domestic academic databases (KoreaMed, KMbase, and KoMGI) were searched for domestic guidelines. The search was limited to the period from 2000 to September, 2023.

An extensive database search was conducted using the following keywords: “tooth fracture”, “radiography”, “crack”, “guideline”, “recommendation” and “cone-beam computed tomography”. The domestic academic databases were searched for domestic guidelines. The working group reviewed the search strategy and results, supplemented by a manual search to ensure that no important guidelines were missed.

### Selection of searched guidelines

Two members of the working group independently reviewed the retrieved literature based on the selection criteria. To ensure objectivity, both primary and secondary screening were performed. Primary screening comprised review of the title and abstract of the study/guidelines, while secondary selection, comprised full text reviews. In cases of disagreement between reviewers, clinical guidelines were selected by consensus. After this selection process, the working group selected the relevant literature and recorded the reasons for exclusion [15, 16].

Inclusion criteria [15, 16]: (1) Clinical guidelines that included PICOs aligned with the key question, (2) Clinical guidelines published in Korean or English, and (3) clinical guidelines published since 2000 were selected.

Exclusion criteria [15, 16]: (1) guidelines do not target patients related to the key question, (2) guidelines not including the relevant imaging modality, (3) guidelines not reporting appropriate outcomes (diagnostic accuracy/efficacy/safety/prognostic impact/patient assessment), (4) non-clinical guidelines, (5) guidelines not presenting recommendations, (6) guidelines not created with evidence-based methods, (7) guidelines reported in languages other than English/Korean, (8) duplicate articles, and (9) full-text not available.

### Search for recent studies

Recent randomized controlled trials or observational studies were searched to ensure that the recommendations were current and reliable. The search period was limited to after the publication date of the most recent guidelines among those selected.

### Quality assessment

The final selected guidelines underwent a rigorous quality appraisal following the Korean Appraisal of Guidelines for Research and Evaluation II (K-AGREE II) [17]. Two reviewers from the development committee assessed the selected literature, with each category scored on a scale of 1–7. To ensure reproducibility and clarity, the reasons for assigning scores were recorded. If the difference in scores between the reviewers was more than four points, the literature was re-examined.

Guidelines that scored ≥ 50 in the “Rigor of Development” domain were considered candidates for K-CIG development [15, 16]. Exceptionally, even documents scoring below 50 points were selected as guidelines for summarizing recommendations and evidence if they were notably scarce or domestically developed clinical guidelines [15].

### Grading the evidence level and drafting the recommendation document

Upon completion of the guideline assessment, recommendations and their supporting evidence were organized using key questions, after which the recommendations were drafted. The table for comparison of guidelines outline the details of the recommendations. In addition, it also assessed whether the guidelines can be accepted and applied in our society.

An evidence table comprising the primary studies included in the selected guidelines was created for each key question. Data from the primary studies were extracted according to a predefined format. The quality of the studies was assessed at the individual study level, and the results were recorded in the evidence table. This process was conducted independently by the two authors, following an agreement process. The evidence levels from individual articles were aggregated to produce an overall evidence level for each recommendation, categorized as high (I), moderate (II), low (III), or very low (IV) [15, 16].

Draft recommendations comprised a recommendation for a key question and a summary of the evidence, considerations, and references, each including a recommendation grade and an overall evidence level. First, the contents of the recommendations related to each key question and the unique recommendation grades were summarized. The recommendation grades were categorized as A (recommendation to implement), B (recommendation to implement under certain conditions), C (recommendation not to implement), and I (no recommendation). The recommendation grade indicates the direction of the recommendation and the evidence level indicates the strength of the recommendation. A comprehensive evaluation was conducted to ensure that the recommendations were updated, acceptable, and applicable, gauging their practicality in the domestic context.

### Agreement of the recommendation grade

The Development Committee reviewed the recommendation grade and evidence level of the draft version of the recommendations created by the working group. The agreed-upon results of the working group and development committee facilitated the definitive assessment of the validity of the recommendation document.

### Finalizing the recommendation document

The consensus group used the Delphi method of anonymity to achieve formal agreement. The first questionnaire included key questions, draft recommendations, recommendation grades, and evidence levels, enabling quick and comprehensive assessment. The agreement level for each recommendation, recommendation grade, and evidence level were assessed and rated on a scale of 1 (strongly disagree) to 9 (strongly agree).

After the first survey, a second questionnaire assessing the level of agreement and opinion was constructed, and a survey was conducted. In the second survey, the distribution of all the respondents in the first survey and each reviewer’s assessment results were provided item-by-item. The reviewer was then asked to decide whether to change or retain the results of the first assessment. An agreement was reached through these iterative rounds.

## Results

### PICO

The PICO guidelines were developed based on key questions generated by the working group. The PICO framework for the key questions is shown in Table 1.

**Table 1:** PICO of the key question

Population	Intervention	Comparators	Outcome
Patients with pain when chewing and suspected VRFs	CBCT	Periapical radiographs	Diagnostic Possibility of vertical root fracture

### Search for guidelines

Tables 2, 3, 4 show the results from international academic databases. Table 5 presents the domestic database results. The KGC and KoMGI yielded no results. All searches were limited to the period from 2000 to September 2023; however, due to NGC being taken off-line in July 2018, searches for NGC were limited to the period up to June 2018.

**Table 2:** Search results from international databases: Ovid-Medline

Searching Date: 2023.9		
*N*	Search Term	Search Result
P (Population)		
1	exp Tooth Fractures/	6609
2	((teeth or tooth or root) and (fracture$ or crack$ or injur$)).mp	40,104
3	1 OR 2	40,104
C (Comparators)		
4	exp Cone-Beam Computed Tomography/ OR CBCT.mp	19,685
5	(intraoral radiography OR tube-shift OR SLOB).tw	403
6	(imaging or radiolog$ or radiograp$).tw	1,452,156
7	OR/4–6	1,463,161
P & C		
8	3 AND 7	4,865
Guideline filter		
9	(guideline$ or recommendation$).ti. or (practice guideline or guideline).pt	159,155
Generalization		
10	8 AND 9	11

**Table 3:** Search results from international databases: Elsevier Embase

Searching Date: 2023.9		
*N*	Search Term	Search Result
P (Population)		
1	‘tooth fracture’/exp	7826
2	((teeth or tooth or root) and (fracture* or crack* or injur*)):ab,ti	32,702
3	1 OR 2	35,865
C (Comparators)		
4	‘cone beam computed tomography’/exp OR CBCT:ab,ti	33,894
5	(‘intraoral radiography’ OR ‘tube-shift’ OR SLOB):ab,ti	420
6	(imaging or radiolog* or radiograp*):ab,ti	2,016,336
7	OR/4–6	2,035,568
P & C		
8	3 AND 7	4714
Guideline filter		
9	guideline*:ti OR recommendation*:ti	186,154
Generalization		
10	8 AND 9	8

**Table 4:** Search results from international databases: GIN, NGC

Searching date: 2023.9 (GIN), 2018.6 (NGC)			
Database	*N*	Search Term	Search Result
1.GIN	1	Tooth Fractures	1
2.NGC	1	Tooth AND Fractures	4

**Table 5:** Search Results from Domestic Databases

Searching Date: 2023.9			
Database	*N*	Search Term	Search Result
1.KoreaMed	1	“Tooth Fractures”[ALL] AND Guideline[ALL]	0
	2	teeth[ALL] and fracture*[ALL] and Guideline[ALL]	2
	3	tooth[ALL] and crack[ALL] and Guideline[ALL]	0
	4	Sum	2
	5	After omitting overlapped literatures	2
2.KMBASE	1	([ALL = vertical root fracture] AND[ALL = recommendation])	0
	2	([ALL = tooth] AND[ALL = fractures] AND[ALL = recommendation])	0
	3	Sum	0
	4	After omitting overlapped literatures	0

### Selection of searched guidelines

**Fig. 1:**
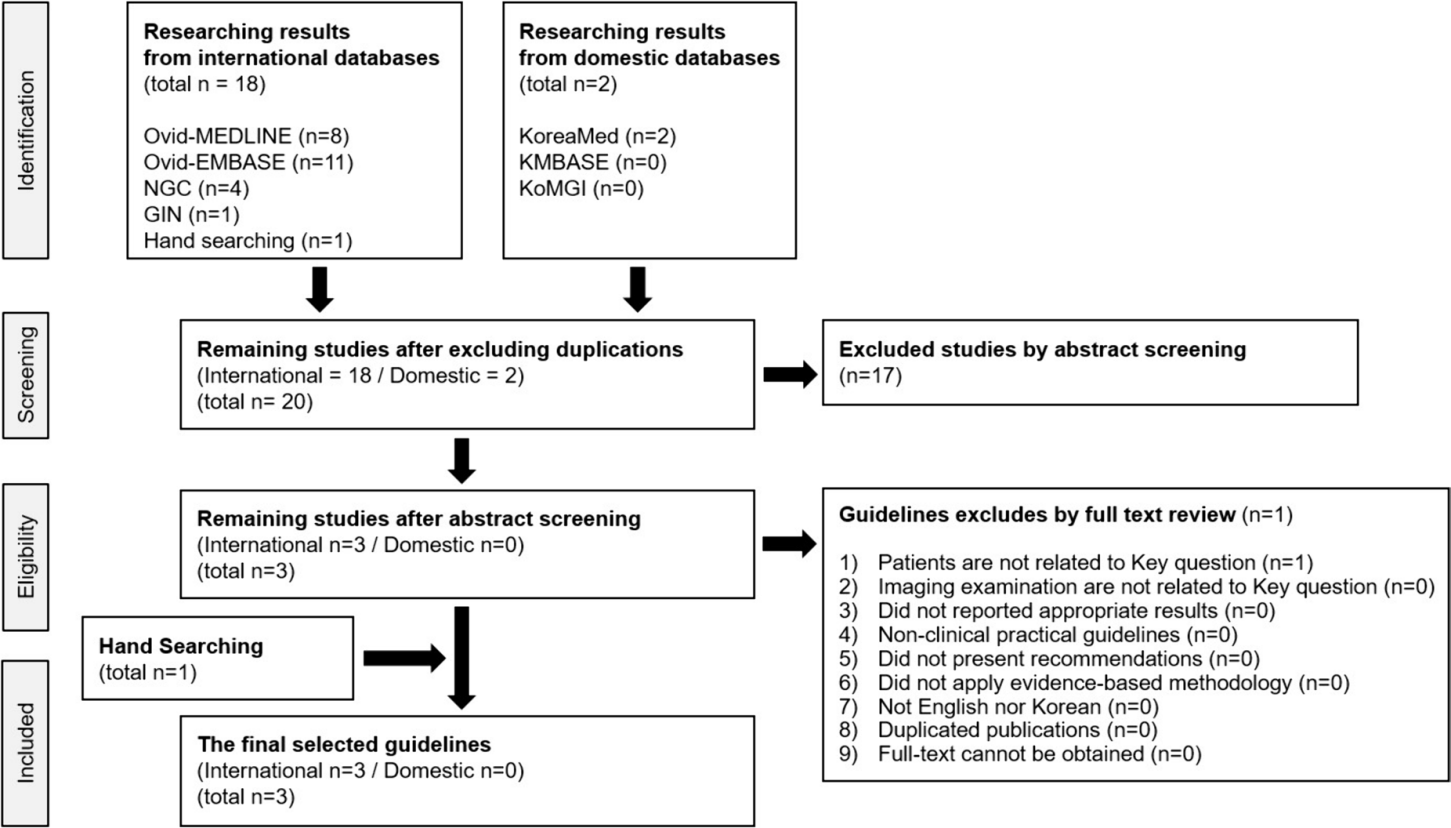
Selection process of searched guidelines

A total of 20 guidelines were retrieved. In the first screening based on titles and abstracts, 17 guidelines were excluded. Secondary selection based on full texts resulted in the exclusion of one additional guideline. However, the manual search identified one more guideline. Consequently, three guidelines were included (Fig. 1). Table 6 presents the list of retrieved guidelines, indicating the selection status and the reasons for exclusion, if applicable.

**Table 6:** Screening result of the searched guidelines

No	Author	Title	Exclusion criteria	
			1st Screening	2nd Selection
1	P. S. Owtad et al. 2015 [18]	Management Guidelines for Traumatically Injured Teeth during Orthodontic Treatment	1	–
2	L. A. S. Kullman 2012 [19]	Guidelines for dental radiography immediately after a dento-alveolar trauma, a systematic literature review	Selected	Selected
3	S. L. Farook et al. 2013 [20]	Guideline for management of hypochlorite injury in endodontics	1	–
4	V. A. Petrosyan, P. 2013 [21]	Third molar surgery: changes in patient demographics over 18 years and the effect of the nice guidelines	1	–
5	M. L. Konishi et al. 2012 [22]	Important technical parameters are not presented in reports of intraoral digital radiography in endodontic treatment: Recommendations for future studies	Selected	1
6	Y. F. Zadik et al. 2008 [23]	Dentists' knowledge and implementation of the 2007 American Heart Association guidelines for prevention of infective endocarditis	1	–
7	A. M. Subbiya et al. 2017 [24]	Radix distolingualis: a case report, review and endodontic treatment guidelines dentistry section	1	–
8	Soares P.B.F. 2020 [25]	Lateral Luxation of Incisor—A Case Report of Using a New Cone-Beam Computed Tomography Software and Reposition Guideline	1	–
9	Lee A.H.C. 2021 [26]	Cemental tear: literature review, proposed classification and recommendations for treatment	1	–
10	Mehrabi F. 2021 [27]	International Association for Dental Traumatology guideline updates	1	–
11	Krastl G. 2022 [28]	Traumatized teeth: clinical practice guideline for the interim management of teeth with various poor prognosis scenarios in growing patients	1	–
12	Hirschhaut M 2023 [29]	Clinical Guidelines for the Surgical/Orthodontic Management of Impacted Maxillary Central Incisors Based on a Decision Tree	1	–
13	Parsons MS 2022 [30]	ACR Appropriateness Criteria R Imaging of Facial Trauma Following Primary Survey	1	–
14	C. National Guideline 2013 [31]	HealthPartners Dental Group and Clinics third molar guideline	1	–
15	C. National Guideline 2017 [32]	Use of adjuvant bisphosphonates and other bone-modifying agents in breast cancer: a Cancer Care Ontario and American Society of Clinical Oncology clinical practice guideline	1	–
16	C. National Guideline 2014 [33]	Perioperative protocol. Health care protocol	1	–
17	C. National Guideline 2013 [34]	ACR Appropriateness Criteria&reg; headache	1	–
18	J.W. KIM et al. 2005 [35]	lifetime and fracture patterns of NITI rotary files in molars	1	–
19	B.S.Kang et al. 2002 [36]	tooth injuries in the emergency department	1	–
20	K. HORNER et al. 2012 [37]	Radiation No 172 Cone beam CT for dental and maxillofacial radiology (Evidence-based guidelines)	Selected	Selected
21	S.M. Mallya 2015 [38]	Evidence and Professional Guidelines for Appropriate Use of Cone Beam Computed Tomography	–	Selected **HAND SEARCH**

Exclusion criteria: (1) guidelines do not target patients related to the key question, (2) guidelines not including the relevant imaging modality, (3) guidelines not reporting appropriate outcomes (diagnostic accuracy/efficacy/safety/prognostic impact/patient assessment), (4) non-clinical guidelines, (5) guidelines not presenting recommendations, (6) guidelines not created with evidence-based methods, (7) guidelines reported in languages other than English/Korean, (8) duplicate articles, and (9) full-text not available

### Search for recent studies

Considering the publication years of the selected recommendations (2011, 2012, and 2015), we searched for studies published between 2015 and September 2023, enrolling those containing evidence current and relevant to the key question or which contributed new insights warranting an update to the content of the evidence table. Studies with designs at the top of the evidence pyramid (meta-analyses > systematic review > cohort studies > case-controlled studies) were primarily selected [39]. Finally, 22 studies were included.

### Quality assessment

Table 7 presents the results of the quality assessment of the three guidelines included in this review using the AGREE II tool [17]. Notably, only one of the three guidelines achieved a score > 50 in the “Rigor of development” domain. However, even lower-scoring guidelines were deemed acceptable in the acceptability/applicability assessment. Because of the limited number of guidelines available to address the key question, these guidelines were included based on the criteria adopted by Choi et al. [15]. Tables 8 present the recommendation matrix and acceptability and applicability assessments of the three guidelines.

**Table 7:** Results of the Quality Assessment of the Guidelines using AGREE II Tool

Title of Guidelines	AGREE Score	Committee Opinion
Guidelines for dental radiography immediately after a dento-alveolar trauma, a systematic literature review	33	Not recommended
Radiation No 172 Cone beam CT for dental and maxillofacial radiology (Evidence-based guidelines)	90	Recommended
Evidence and Professional Guidelines for Appropriate Use of Cone Beam Computed Tomography	31	Not recommended
Not recommended: AGREE II < 50

**Table 8:** Summary of recommendations selected from the existing guidelines and used to development

	Guideline A	Guideline B	Guideline C
Recommendation	A suspicion of a vertical root fracture establishes a need for different horizontal angulations to be used during exposure and a transverse fracture needs different vertical angulations. CBCT could be an acceptable option in special cases, where there is a problem to find a clinically suspicious root fracture (minimally displaced root fractures)	Limited volume, high resolution CBCT is indicated in the assessment of dental trauma (suspected root fracture) in selected cases, where conventional intraoral radiographs provide inadequate information for treatment planning	CBCT imaging should be used only when the diagnostic information is not provided by conventional intraoral radiography and when the additional information from CBCT is likely to aid diagnosis and treatment planning Second, as with all other imaging, the decision to pre-scribe a CBCT scan must be based on the patient’s history and clinical examination and justified on an individual basis. CBCT imaging can be used to augment clinical examination and conventional radiography in complex endodontic conditions
Grading of recommendation	Not Available	B	Not Available
Aceeptability			
Similarity of population	uncertain	uncertain	uncertain
Similarity of value and preference	Yes	Yes	Yes
Similarity of benefit by recommendation	Yes	Yes	Yes
Generally, acceptable	Yes	Yes	Yes
Applicability			
Applicability of intervention/instrument	Yes	Yes	Yes
Applicability of essential technique	Yes	Yes	Yes
No legal and institutional barriers	Yes	Yes	Yes
Generally, applicable	Yes	Yes	Yes

Guideline A: Guidelines for dental radiography immediately after a dento-alveolar trauma, a systematic literature review [19]

Guideline B: Radiation No 172 Cone beam CT for dental and maxillofacial radiology (Evidence-based guidelines) [37]

Guideline C: Evidence and Professional Guidelines for Appropriate Use of Cone Beam Computed Tomography [38]

### Grading the evidence level and drafting the recommendation document

Recommendations were proposed based on the three reviewed guidelines. Subsequently, an evidence table was prepared by summarizing the individual articles relevant to the key question and assigning an evidence level (Table 9). The proposed recommendations, recommendation grades, and evidence levels are as follows:

**Table 9:** Evidence Table

Author, Year	Title	Type of Study	Patients (*n*)	Study Quality (KCIG)
Wenzel et al. 2005 [40]	High resolution charge-coupled device sensor vs. medium resolution photostimulable phosphor plate digital receptors for detection of root fractures in vitro	Experimental	47 teeth	1
Kamburoglu et al. 2009 [41]	Effectiveness of limited cone-beam computed tomography in the detection of horizontal root fracture	Experimental	36 teeth	2
Bernardes et al. 2009 [42]	Use of cone beam volumetric tomography in the diagnosis of root fractures	Experimental	20	1
Hassan et al. 2009 [7]	Detection of vertical root fractures in endodontically treated teeth by a cone beam computed tomography scan	Experimental	80 teeth	2
Őzer et al. 2010 [3]	Detection of vertical root fractures of different thicknesses in endodontically enlarged teeth by cone beam computed tomography versus digital radiography	Experimental	80 teeth	2
Varshosaz et al. 2010 [43]	Comparison of conventional radiography with cone beam computed tomography for detection of vertical root fractures: an in vitro study	Experimental	100 teeth	2
Kamburoğlu et al. 2010 [44]	Detection of vertical root fracture using cone-beam computerized tomography: an in vitro assessment	Experimental	60 teeth	2
Hassan et al. 2010 [45]	Comparison of five cone beam computed tomography systems for the detection of vertical root fractures	Experimental	80 teeth	2
Edlund et al. 2011 [46]	Detection of vertical root fractures by using cone beam computed tomography: a clinical study	Observational	32 teeth	2
Wanderley et al. 2018 [47]	Influence of Tooth Orientation on the Detection of Vertical Root Fracture in Cone-beam Computed Tomography	Experimental	30	2
Ezzodini et al. 2015 [48]	Diagnostic value of cone-beam computed tomography and periapical radiography in detection of vertical root fracture	Experimental	80 teeth	3
Salineiro et al. 2017 [49]	Radiographic diagnosis of root fractures: a systematic review, meta-analyses and sources of heterogeneity	Review	47 studies	2
Amintavakoli et al. 2017 [50]	Reliability of CBCT diagnosing root fractures remains uncertain	Review	12 studies	3
Kobayashi et al. 2017 [51]	Diagnosis of alveolar and root fractures: an in vitro study comparing CBCT imaging with periapical radiographs	Experimental	75 teeth	3
Talwar et al. 2016 [52]	Role of Cone-beam Computed Tomography in Diagnosis of Vertical Root Fractures: A Systematic Review and Meta-analysis	Review	11 review 4 meta	1
Chang et al. 2016 [4]	Cone-beam Computed Tomography for Detecting Vertical Root Fractures in Endodontically treated Teeth: A Systematic Review	Review	4 studies with a total of 130 patients	2
Ma et al. 2016 [53]	RH, Ge ZP, Li G. Detection accuracy of root fractures in cone-beam computed tomography images: a systematic review and meta-analysis	Review	13 studies	1
Habibzadeh et al. 2023 [54]	Diagnostic efficacy of cone-beam computed tomography for detection of vertical root fractures in endodontically treated teeth: a systematic review	Review	20 studies	2
de Lima et al. 2023 [55]	Influence of the technical parameters of CBCT image acquisition on vertical root fracture diagnosis: a systematic review and meta-analysis	Review	60 studies 60 reports	2
PradeepKumar et al. 2021 [56]	Diagnosis of Vertical Root Fractures by Cone-beam Computed Tomography in Root-filled Teeth with Confirmation by Direct Visualization: A Systematic Review and Meta-Analysis	Review	8 studies	2
Al Hadi et al. 2020 [57]	Detection of Vertical Root Fractures Using Three Different Imaging Modalities: An In Vitro Study	Experimental	60 teeth	2
Hekmatian et al. 2018 [58]	Detection of Vertical Root Fractures Using Cone-Beam Computed Tomography in the Presence and Absence of Gutta-Percha	Experimental	50 teeth	2


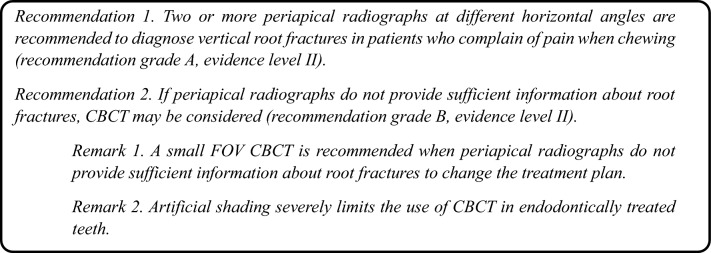


### Finalizing the recommendation document

The Delphi method was used to ensure data anonymity. The recommendation document was finalized after two rounds of evaluation by six experts from relevant clinical fields. The level of agreement was quantified, yielding means of 8.0 (SD 1.2) and 7.6 (SD1.6) for Recommendations 1&2 (Table 10).

**Table 10:** Result of Delphi method

Recommendation No	Recommendation grade	Evidence Level	Average	MIN	Q1	Median	Q2	MAX	SD	CV	Number of respondents
1	A	II	8.0	6	7.5	8.0	9.0	9	1.2	0.1	6
2	B	II	7.6	5	6.5	8.0	9.0	9	1.6	0.2	6

*MIN* minimum, *Q* Quartile, *MAX* maximum, *SD* standard deviation, *CV* coefficient of variation

## Discussion

Herein, we formulated imaging guidelines for diagnosing VRF based on three guidelines and 22 evidence-based studies. The literature suggests the primary use of two-dimensional radiographs, reserving CBCT for cases difficult to diagnose using PA radiography. As with other radiologic modalities, CBCT should only be used when the benefits outweigh the risks [12, 13]. Therefore, the dentists should ensure that the information obtained from CBCT imaging can enhance patient care, patient safety, and ultimately enable more predictable and optimal treatment [59].

To detect vertical and horizontal root fractures, Wenzel et al. emphasized the necessity of obtaining PA radiographs at two and three vertical and horizontal angles, respectively. Specifically, a minimum of three PA radiographs were required, each differing by 15°, vertically and horizontally [40]. Other studies suggest aligning the X-ray in 2D radiography parallel to the fracture line (± 4°) for optimal diagnostic accuracy [60]. In addition, the visibility of the fracture line is influenced by the degree of displacement or separation of the fragments [61]. The radiologic features of VRF are as follows: a visible fracture line, separation of root fragments, space between the root filling and the canal wall, vertical bone loss, and characteristic diffused or halo/J-type radiolucency around the root [48].

A previous study showed that conventional radiographs have low sensitivity for detecting minimally displaced root fractures [61]. Although CBCT offers improved sensitivity, it is expensive and inappropriate for routine use due to the high radiation dose [41]. When diagnosing VRF, an inexpensive method that minimizes radiation exposure and is readily clinically applicable, such as PA radiography, is required. In untreated teeth, when both clinical and digital radiological data are insufficient, CBCT may be appropriate to identify VRF [57].

CBCT allows precise visualization and evaluation of teeth with VRFs, with a higher sensitivity, specificity, and accuracy than PA radiography [5, 7]. One in vitro study found significantly elevated sensitivity in CBCT imaging compared to PA (0.752 vs 0.242) when using a 0.2-mm voxel size to detect VRFs in unfilled teeth [52]. Another study used nine differently angled images of each tooth and reported a low sensitivity (28%) and specificity (33%) for conventional 2D images, while CBCT imaging showed a higher sensitivity (55%) and specificity (82%) [6]. The heightened sensitivity of CBCT compared to PA radiography stems from its capacity to provide multi-planar views at various angles and orientations, utilizing extremely thin slices and high contrast [52].

Given that VRFs are predominantly associated with endodontically treated teeth, evaluating the potential impact of root canal filling on fracture line visibility is crucial [7]. The diagnostic odds ratio (DOR) serves as a metric of the effectiveness of a diagnostic test. In one study, CBCT demonstrated superior performance in unfilled teeth (DOR = 94.26) compared to PA radiography (DOR = 14.42) [52]. However, in filled teeth, no significant difference was observed between the collective DOR, with PA radiography slightly outperforming CBCT [52]. The mean (SD) sensitivity (%) of CBCT for detecting VRFs in the presence of gutta-percha and metal posts was 72.76 (18.73), ranging from 30 to 92%, while the mean specificity (%) was 75.44 (18.26), ranging from 45 to 100% [54].

The lower sensitivity of CBCT in root-filled teeth may be attributed to unique challenges, such as beam hardening and artifact generation [52]. Given the prevalence of VRFs in root-filled teeth [58], these artifacts may resemble root fractures or overlapping root fracture lines, leading to incorrect diagnoses [62].

However, recent studies have reported conflicting results. Al Hadi et al. [57] reported that root canal filling material did not significantly affect the specificity of VRF detection on CBCT images (100% in both groups). Similar outcomes were observed in three other recent studies [63–65]. These results can be attributed to the enhanced image resolution generated by the CBCT systems used [57]. The researchers concluded that image quality was directly affected by voxel size in CBCT examinations, recommending a 0.2–0.3-mm voxel resolution scan to diagnose VRF [64]. On the other hand, other recent studies still question the usefulness of CBCT in diagnosing VRF in obturated teeth. Patel et al. [66] and Chang et al. [4] each concluded in their in vitro and systematic reviews that using CBCT to detect VRF in root-filled teeth is inaccurate.

The disparate outcomes presented in recent studies may be due to variations in the image quality and performance of the CBCT systems. Elsaltani et al. [63] reported that the accuracy of VRF diagnosis in endodontically treated teeth varied based on the CBCT system used, with the smallest voxel size of 0.125 mm (i-CAT) exhibiting superior accuracy. However, voxel size alone is insufficient for diagnostic accuracy, as different systems with the same 0.2-mm voxel size (Planmeca ProMax 3D, J Morita, and Galileos 3D) demonstrated varying accuracies [63]. Another factor affecting diagnostic accuracy is the type of detector used. Instruments with high diagnostic capabilities use flat panel detectors, whereas those using image intensifier tubes or charge-coupled devices have relatively low diagnostic capabilities [63].

Nonetheless, the appropriate imaging modality for VRF may vary depending on the location of the tooth (maxillary/mandibular, anterior/posterior) or the number of roots. This guideline is based on existing guidelines and research papers, necessitating foundational research. However, due to a lack of supporting literature, these factors have not been included in this guideline. As the guideline will be updated periodically, if research results reflecting these aspects are accumulated, it is likely that more specific guidelines can be established in the next update.

When developing and applying the proposed guidelines, cost considerations must also be taken into account. The costs of imaging tests and the extent of health insurance coverage can vary by country. In South Korea, PA and CBCT are often covered by insurance. These factors were considered in the assessment of acceptability and applicability. However, even if the cost of CBCT is not prohibitively expensive and it can be performed without difficulty, the periapical view, which can diagnose with lower radiation exposure and cost, should be the first choice.

Compared to traditional radiography, CBCT slightly increases the patient’s radiation exposure to yield more comprehensive information. Therefore, in most cases, an increased radiation dose is justifiable. However, the unwarranted use of CBCT raises concerns as conventional plain films can fulfill many diagnostic requirements with a lower radiation dose [43]. The radiation dose in CBCT remains higher than that in traditional panoramic imaging systems. According to the 2007 International Commission on Radiological Protection guidelines, the effective radiation doses calculated for i-CAT with 6- and 13-inch FOVs are 75.3 and 110.5 mSv, respectively, which are 5.8–8.5 times higher than panoramic imaging (13 mSv) [67]. Generally, methods with higher sensitivity are preferred. However, given the high radiation dose and cost associated with CBCT, and the high specificity of PA radiography (low false-positive rate), CBCT confirmation is deemed unnecessary if VRF are observed on PA radiography. Nevertheless, CBCT is recommended when VRF are suspected and PA radiography fails to detect them [48].

Recent studies have focused on developing techniques to reduce the exposure dose of CBCT while maintaining image quality. Loubele et al. investigated the correlation between the radiation dose and image quality of four CBCT scanners, concluding that high-resolution i-CAT exhibited the most favorable image quality to radiation dose ratio [68]. Another proposed method, by Mora et al. [69], involved reducing the number of basis projections. Following a previous study investigating the accuracy of limited cone-beam computed tomography for vertical fracture detection [5], a subsequent study [69] revealed that a significant reduction in radiation exposure could be achieved by decreasing the number of images from 180 to 60 without a difference in root fracture diagnosis. As CBCT continues to gain popularity, further studies are required in this emerging field [43].

This guideline was created through a systematic review, end-user review, and consensus. The selective use of PA and CBCT will serve as a valuable resource for clinicians deciding on appropriate imaging modalities VRF diagnosis.

## Conclusion

This study developed evidence-based clinical imaging guidelines for the diagnosis of VRFs. The applicability of these guidelines should be consistently monitored and evaluated to ensure the best possible patient outcomes in a clinical setting.

## Data Availability

The data analyzed during the current study will be available from the corresponding author upon reasonable request.

## Declarations

### Human or animal subjects

This article does not contain any studies with human or animal subjects performed by the any of the authors.
